# Supplementary material for: Comprehensive Annotation and Functional Exploration of MicroRNAs in Lettuce
Source: Front Plant Sci. 2021 Dec 24;12:781836. doi: 10.3389/fpls.2021.781836 (PMC8739914; doi:10.3389/fpls.2021.781836)
Supplement: Supplementary file 2 [file Data_Sheet_8.ZIP › GSM2230747.plot/Lsa-miR398b_Lsat_1_v5_gn_4_153121.1_509_TPlot.pdf]

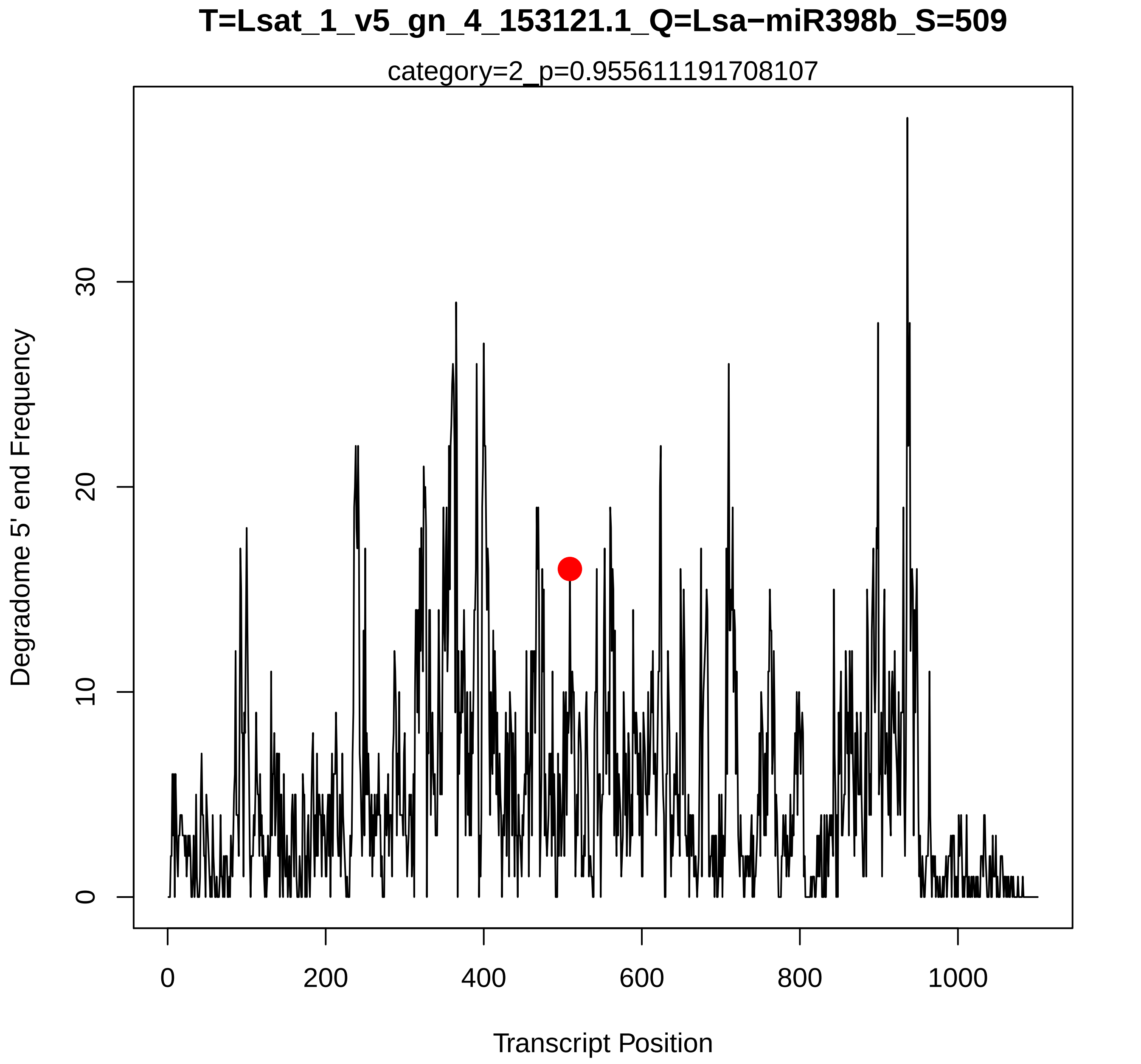
T=Lsat_1_v5_gn_4_153121.1_Q=Lsa−miR398b_S=509
category=2_p=0.955611191708107
Degradome 5' end Frequency
0
10
20
30
0
200
400
600
800
1000
Transcript Position
